# On Epilepsy and Epileptiform Seizures : Their Causes, Pathology, and Treatment

**Published:** 1858-10-01

**Authors:** 


### REVIEWS

*On Epilepsy and Epileptiform Seizures: their Causes, Pathology, and Treatment.* By Edward Henry Sieveking, M.D., Physician to St. Mary's Hospital, &c. (Churchill, 1858.)

That dire disease which ever and anon suspends reason and convulses the frame—which strikes with the suddenness of lightning and with the awfulness of unseen power, has always been a subject of intense interest to the medical inquirer. The psychological physician especially has been impelled to study it for two reasons. First, on account of the intimate etiological connexion of epilepsy with mental alienation; secondly, because a large proportion of the victims of this disease, driven from society, become permanent burdens upon the public and private asylums. He is moved by the spirit of philosophical investigation to inquire into the pathology of epilepsy; and stimulated by the practical necessity of governing a body of epileptics to seek the means of alleviating or of curing the disease. Accordingly, we find that some of the most elaborate and valuable treatises on epilepsy have emanated from special psychologists. It is certainly not our desire to detract one iota from the sterling merit or usefulness of such contributions as those of Esquirol, Delasiauve, or Cazauvieilh. But it may be permitted to us to welcome with cordiality and satisfaction the work of a general physician who has studied the disease from a point of view distinct from that of the psychological specialist. There is a peculiar advantage in carrying back our inquiries into the etiology and pathology of epilepsy to the epochs which stand nearest in time to the origin of the disease.

The general physician stands in a position to do this. He witnesses all those earlier phenomena which attend and usher in the outbreak of the malady. He sees its growth, and he is able to watch the circumstances which, for good or for evil, modify its progress. The testimony of the general physician is necessary to complete the history. He alone in fact can read the first chapters, without which the subsequent ones, written in the lunatic asylum, must remain imperfect, unintelligible, and unprofitable.

This consideration would alone be sufficient to give value to the work of Dr. Sieveking. But those who may peruse his work will not fail to acknowledge that its value is enhanced by the excellent qualities which have enabled him to turn his special advantages to the best account. The work extends over 267 pages of small octavo. Not only on account of its moderate bulk, but also from the mass of valuable matter and judicious reflections it contains, Dr. Sieveking's book will be generally studied by the profession. It is not, therefore, necessary to enter into any lengthened analysis. It will be sufficient if we call prominent attention to the leading points which the author has more particularly illustrated.

Dr. Sieveking deprecates exclusive regard to the paroxysm as constituting the essence of epilepsy. He agrees with Dr. Watson in objecting to definitions of epilepsy, because its forms are so various, and its modifications so numerous, that no description can be given of it. He inclines to think that our knowledge of the disease will not gain by such definitions, as they tend to limit the attention to the paroxysm, and often mislead the observer, by inducing him to overlook conditions to which the definition does not apply, but which will appear, after a further examination of the whole subject, to belong to the same category of diseased conditions as epilepsy. We concur with the author thus far, that we should be keeping out of sight important etiological and pathological conditions if we restricted our investigations to the paroxysm itself. By losing sight of these conditions we should also vastly diminish the guides to curative or preventive treatment. Yet the study of the paroxysm is replete with physiological interest, and we will add, with promise of useful therapeutical application. The brilliant light which Brown-Séquard is throwing upon the functions of the nervous system, will probably before long clear up the mystery of the immediate cause of the epileptic seizure. When we shall have attained this point, an immense gain will have been made. The remarkable, we may say the cardinal, experiments of this far-sighted physiologist tend to define more closely the individuality of the function of the various parts of the nervous system. There can be no doubt that the epileptic seizure depends primarily upon a particular condition of a particular part, perhaps a very small part, of the nervous centres. It will be by the careful comparison of Nature's experiments upon the human subject with the physiologist's experiments upon the lower animals, that we shall one day demonstrate the essence of the epileptic paroxysm. It must, moreover, not be forgotten that the physiological analysis of the epileptic paroxysm has already led Marshall Hall to the discovery of the means of cutting-off one of the most disastrous elements of the paroxysms, and thus of making one grand step on the road towards its total abolition. We do not fully assent to the truth of the flowery metaphor of Dr. Sieveking, and "regard the fit as (simply) the flower of a noxious weed." We rather regard the fit as the essence of the disease; but that, like many other diseases, the fit may be produced by a variety of causes. Many roads may lead to the same goal. But difference in the mode of production and variation in the route do not change the identity of the result. For these reasons we would continue to fasten our attention upon the fit as the foremost object of study; not, be it well understood, neglecting the close observation of those conditions which precede and determine its outbreak.

In discussing the paroxysms, Dr. Sieveking appears to question their subjection to the law of periodicity. He says:—

"Neither in my own observations, nor in the histories of the disease preserved by other authors, has there appeared to be any uniformity in the mode in which the paroxysms took place. In many, granted even the majority of cases, no regularity of return can be detected. But certainly our own observation affords distinct examples of periodicity. A young gentleman, destined for the Dutch navy, was seized with his first epileptic paroxysm on board-ship; a second occurred just one month, less twenty-four hours, after; he noted this interval, and being sent to London to be placed under our care, declared his expectation that another attack would occur after the like interval. He was domiciled in our own house. A fit of a severe character, lasting nearly an hour, seized him at the very moment he had predicated. On the return of the next period, the ruse was resorted to of putting the clock forwards; nevertheless, and although he was cheerful up to the moment, not apparently meditating the attack, he was suddenly arrested in an occupation which appeared to engross his mind, and at a time when he had reason to believe his hour was past, by the paroxysm. This periodicity exhibited itself on other occasions. It was never disturbed until he was altogether cured, an event which took place in a few months. Again, a gentleman engaged in an arduous commercial pursuit was subject to a cataleptic suspension of consciousness, the *petit mal, every Sunday*. It occasionally attacked him at other times, but on the Sunday it never failed until he was put under treatment. Again, we have now under our care a sailor, in an institution, where constant observation is exercised, who has three several times had a fit at as nearly as possible intervals of thirty days. We might multiply such examples. We cannot therefore doubt the fact of a periodical tendency. We even see in this tendency an important element towards the pathological elucidation of the disease."

Some excellent chapters follow on the phenomena and causes of epilepsy. The pathological anatomy is treated with especial care. The author dwells particularly on the observations of Dr. Boyd, which show an increased weight of brain in epileptics; he analyses at some length the less known researches of Wenzel, who seeks to place the pathological seat of epilepsy in the pituitary body and pineal gland. He does justice to this observer by correcting a misapprehension which has attached to his views; he repeats the warning before pointed out by Dr. Sims, not to confound, as has been done, the *cerebellum* with the pituitary body; this error he conjectures to have arisen from the use of the French translation of Wenzel, which gives the title "*Cervelet*" for "*Hirnanhang*." Dr. Sieveking, although considering the bearing of disease of the pituitary body upon epilepsy, like that of Addison's disease to the bronzing of the skin, as a moot point, yet attaches great importance to the completeness and accuracy of Wenzel's twenty autopsies of epileptics. He gives a summary of each case.

The chapter on the theory of epilepsy is well deserving of attention. Dr. Sieveking's mode of discussing the value of different facts and hypotheses is eminently philosophical. His appreciation is that of a calm, reflective, and unprejudiced critic. He believes that in the great majority of instances of epilepsy, the first attack is due to an irritation produced by derangement in the amount or quality of the blood circulating in the brain. In a person predisposed we frequently find over-fatigue, a long walk, carrying heavy loads, or prolonged mental exertion, the manifest cause not only of the first, but of many succeeding seizures. Hence there will be occasion, in discussing the treatment of the disease, to dwell much upon the necessity of bodily and mental rest, so as to allow the system to recover that balance the disturbance of which gave rise to the seizure.

The two concluding chapters are devoted to the treatment of epilepsy. They exhibit, equally with the preceding ones, the quiet sagacity and excellent judgment of the author. Consistently with his view, that the general condition of the patient rather than the paroxysm should be regarded, he points out the special importance of treating the patient for the intervallic phenomena. It is here, again, that the experience of the general physician is useful in correcting or completing the experience of the psychiatric specialist. Esquirol has declared an absolute scepticism as to the influence of remedies. But the remark of Dr. Sieveking is true, that a lunatic asylum is generally made the ultimate resort of epileptic patients in whom the usual remedies have been exhausted, and in whom incipient mental fatuity has already indicated organic intra-cranial lesion. The experience of Dr. Sieveking certainly agrees with our own, namely, that medicine, taking a large view of the term, is capable of effecting much. "If I were to formularize" (says Dr. Sieveking) "the prevailing mode of treatment which I myself adopt, I should say it consisted in local derivation, or counter-irritation directed against cerebral congestion, and in general roborants or tonics." Of the presumed specific remedies he is disposed to place the preparations of iron and zinc first. Of zinc he would speak very favourably; he prefers the soluble sulphate to the oxide. He was probably unacquainted with the diphosphate, the preparation recently introduced by Dr. Barnes, and recommended by arguments deserving of attention. This skilful practitioner observes, that the combination of zinc with phosphorus seems particularly indicated by the expenditure of phosphorus in brain labour, by the prevalence of phosphorus in grain and many important articles of food; and by the system tolerating better the phosphate than the sulphate. If the object be to get more zinc into the system, phosphoric acid is the better vehicle. In many cases, Dr. Barnes' combination of diphosphate of zinc, quinine, and valerian seems to be especially promising. He speaks slightingly, and our experience agrees with his, of indigo and cotyledon umbilicus.

Passing from specific remedies, Dr. Sieveking dwells with much emphasis on the influence of moral and hygienic treatment. The cold bath is especially extolled.

The book concludes with a tabular summary of fifty-six cases observed by the author. Our notice must conclude with the expression of our thankfulness for so valuable a contribution to the pathology and treatment of this obscure and intractable disease.

